# A computational model of interconnected basal ganglia-thalamocortical loops for goal directed action sequences

**DOI:** 10.1186/1471-2202-12-S1-P136

**Published:** 2011-07-18

**Authors:** Jennifer M Lewis, Jonathan M Chambers, Peter Redgrave, Kevin Gurney

**Affiliations:** 1 Department of Psychology, University of Sheffield, Sheffield, S10 2TN, UK

## 

The basal ganglia (BG) are a group of subcortical nuclei which are believed to be involved in mediating action selection [1]. They are arranged in a series of topographically organised anatomical ‘loops,’ receiving input from large regions of cortex, and projecting back to the same cortical regions via thalamus. These basal ganglia-thalamocortical (BGTC) loops are widely accepted to be largely segregated. However, increasing anatomical evidence suggests that while this general topography holds, there is significant interconnectivity between loops [2]. This results in a predominantly hierarchical flow of information from regions of cortex and BG associated with motivational processing, through cognitive, and finally to motor regions. This organisation suggests a mechanism by which goal related and contextual information may bias processing in circuits associated with motor actions.

We present a model of two BGTC loops which exploits this inter-loop connectivity to integrate several types of information required for the production of goal directed action sequences. An ‘associative loop’ comprising prefrontal cortex (PFC) and its corresponding regions of BG receives information regarding the current goal. By integrating this with stored knowledge of a relevant goal directed action sequence, the loop forms a unique, self-sustaining representation of the temporal task context which indicates the current stage in the ongoing task. A lower ‘motor loop’ is responsible for action selection based on affordance information regarding the objects in the environment available to it. Processing in this loop is influenced by the contextual representation in PFC via corticostriatal connectivity linking the two loops. Under reinforcement learning, these projections adopt the appropriate connectivity to successfully bias contextually appropriate action selection in the motor loop. Changes to the environment as a result of the action selected by the model initiate new representations in PFC, subsequently biasing further action selection, and thereby generating sequential behaviours. The self sustaining nature of prefrontal representations allows the model to function with phasic, rather than sustained feedback signals regarding the action just performed.

Four actions – A, B, C and D – are available for selection. The extended sequences ABACAC and ADCACAC are required to achieve goals 1 and 2, respectively. The model is able to consistently execute the required sequences. These results indicate the utility of the BGTC architecture in generating flexible behaviours, in which component actions may be recruited: i) towards multiple goals; ii) as part of distinct sequences; iii) independently of the action just performed; and iv) multiple times in a single sequence. Observed action switching times of approximately 200ms are comparable with existing primate behavioural and neurophysiological data [3], and are an emergent property of the biologically constrained time constants and architecture employed. These results have significant implications for the proposed integrative function of BGTC interconnectivity, and its role in organising sophisticated, contextually appropriate action selection.
